# 383. Feasibility of Specimen Self-collection in Young Children Undergoing SARS-CoV-2 Surveillance for In-person Learning

**DOI:** 10.1093/ofid/ofab466.584

**Published:** 2021-12-04

**Authors:** Jonathan Altamirano, Grace Tam, Marcela Lopez, India Robinson, Leanne Chun, Nuzhat Shaikh, Sean Leary, Yuan J Carrington, Shilpa Jani, Uma Pulendran, Jasmin Ma, Elizabeth Toomarian, C Jason Wang, Prasanthi Govindarajan, Andra Blomkalns, Makeda Robinson, Yvonne A Maldonado

**Affiliations:** 1 Stanford University School of Medicine, Stanford, CA; 2 Stanford University, Stanford, CA

## Abstract

**Background:**

While pediatric cases of COVID-19 are at low risk for adverse events, schoolchildren should be considered for surveillance as they can become infected at school and serve as sources of household or community transmission. Our team assessed the feasibility of young children self-collecting SARS-CoV-2 samples for surveillance testing in an educational setting.

**Methods:**

Students at a K-8 school were tested weekly for SARS-CoV-2 from September 2020 - June 2021. Error rates were collected from September 2020 - January 2021. Clinical staff provided all students with instructions for anterior nares specimen self-collection and then observed them to ensure proper technique. Instructions included holding the sterile swab while making sure not to touch the tip, inserting the swab into their nostril until they start to feel resistance, and rubbing the swab in four circles before repeating the process in their other nostril. An independent observer timed random sample self-collections from April - June 2021.

**Results:**

2,590 samples were collected from 209 students during the study period when data on error rates were collected. Errors occurred in 3.3% of all student encounters (n=87). Error rates over time are shown in Figure 1, with the highest rate occurring on the first day of testing (n=20/197, 10.2%) and the lowest in January 2021 (n=1/202, 0.5%). 2,574 visits for sample self-collection occurred during the study period when independent timing data was collected (April - June 2021). Of those visits, 7.5% (n=193) were timed. The average duration of each visit was 70 seconds.

Figure 1. Swab Error Rates Over Time

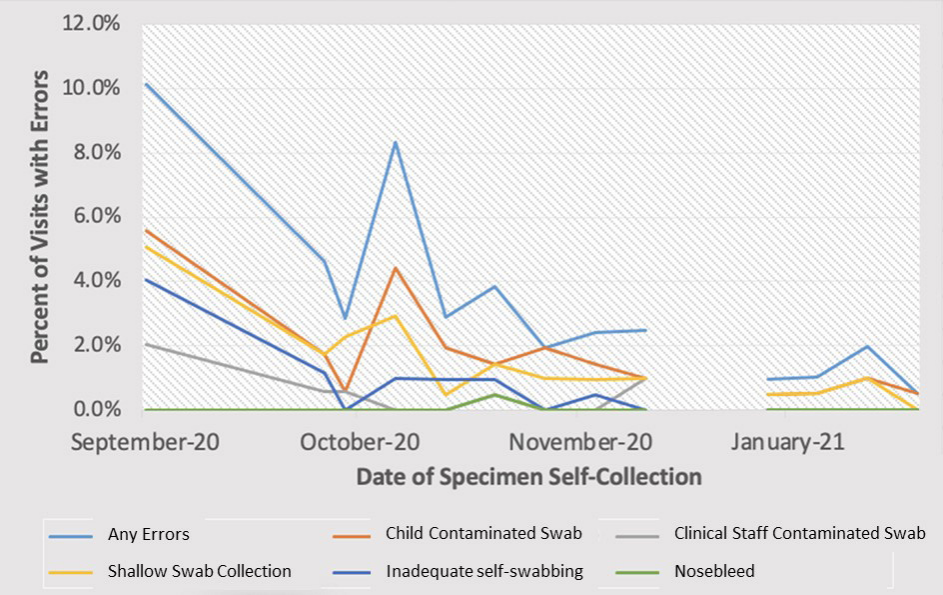

**Conclusion:**

Pediatric self-collected lower nasal swabs are a viable and easily tolerated specimen collection method for SARS-CoV-2 surveillance in school settings, as evidenced by the low error rate and short time window of sample self-collection during testing. School administrators should expect errors to drop quickly after implementing testing.

**Disclosures:**

**All Authors**: No reported disclosures

